# Prevalence and factors associated with unmet need for family planning among women of reproductive age (15–49) in the Democratic Republic of Congo: A multilevel mixed-effects analysis

**DOI:** 10.1371/journal.pone.0275869

**Published:** 2022-10-07

**Authors:** Marie Alice Mosuse, Sylvie Gadeyne

**Affiliations:** Faculty of Economic and Social Sciences & Solvay Business School, Department of Social Research, Interface Demography, Brussels, Belgium; UNITED KINGDOM

## Abstract

The Democratic Republic of Congo (DRC) has experienced high levels of unmet need for family planning (UNFP) for many years, alongside high fertility, maternal and infant mortality rates. Previous research addressed the UNFP in DRC, but analyses were limited to the individual-level and to specific regions. This study aims to determine the individual- and community-level factors associated with UNFP among married women of reproductive age in DRC. Using data from the 2014 DRC Demographic and Health Survey, a two-level mixed-effect logistic model examined i) the associations between UNFP and individual- and community level factors, and ii) the extent to which individual variability in UNFP is due to the variability observed at the community-level, given the individual characteristics. A total of 10,415 women in 539 clusters were included. Prevalence of unmet need for limiting was 8.13%, and 23.81% for spacing. Compared to adolescents (15–24), young (25–34) (aOR = 0.75, CI: 0.63–0.90) and middle-aged (35–49) (aOR = 0.65, CI: 0.51–0.82) women were less likely to have unmet need for family planning. The odds of having unmet need increased significantly with number of living children [1–2 children (aOR = 2.46, CI: 1.81–3.35), 7+ children (aOR = 6.46, CI: 4.28–9.73)] and among women in a female-headed household (aOR = 1.22, CI: 1.04–1.42). Women from provinces Equateur (aOR = 1.82, CI: 1.24–2.68), Nord-Kivu (aOR = 1.66, CI: 1.10–2.55) and Orientale (aOR = 1.60, CI: 1.10–2.32) were more likely to have unmet need, compared to women from Kinshasa. Women from communities with medium (aOR = 1.32, CI: 1.01–1.72) and high (aOR = 1.46, CI: 0.98–2.18) proportion of women in wealthy households, and medium (aOR = 1.32, CI: 1.01–1.72) and high (aOR = 1.46, CI: 0.98–2.18) proportion of women with low ideal family size (≤6) were more likely to have unmet need, compared to those from communities with low proportion of wealthy households and high ideal family size, respectively. Policies should consider strengthening family planning programs in provinces Equateur, Orientale, and Nord-Kivu, and in wealthier communities and communities with a higher ideal family size. Family planning programs should target adolescents and young women.

## Introduction

Unmet need for family planning refers to the discrepancy between fertility preferences and contraceptive usage [1]. Women who have unmet needs want to stop or delay childbearing but are not using any method of contraception [2]. Unmet need reveals the lack of accessible family planning services or knowledge available to women with a need for contraceptives [3]. Unmet need can also be related to other barriers to contraception, such as fear of side effects or health concerns, social acceptability, or cultural restrictions [1,3,4]. Tackling the unmet need for family planning has been a central concern of global health and population policies for decades [5]. Not only because it is related to high fertility and associated social, economic, and environmental challenges, but also because of its link with women’s empowerment [6]. According to the demographic transition theory, high fertility results from the combination of high desired family size and high child mortality [7]. Lowering the fertility preference is therefore a crucial factor to decrease high fertility. For women to act accordingly to the desired family size, they need access to contraception however [6,8]. Enabling women to choose for pregnancy—rather than being compelled—will in turn generate social and economic benefits beyond the health sector: higher educational attainment, increased female labor participation, and greater accumulation of household wealth [8]. In addition, decreasing high fertility reduces future population growth and associated challenges and accelerate progress towards achieving the Sustainable Development Goals [8,9]. Two Sustainable Development Goals are in line with tackling the unmet need for family planning. The third goal (SDG3) aims to ensure healthy lives and promote well-being for all, including the universal access to sexual and reproductive health-care services and the integration of reproductive health into national strategies and programs, and the second target (SDG5) strives for gender equality and the empowerment of women and girls, aiming to ensure universal access to sexual and reproductive health and reproductive rights [9].

Unmet need for family planning is higher in sub-Saharan Africa than anywhere else in the world [3]. Nearly 26% of sub-Saharan African women of reproductive age had unmet need for family planning between 2000 and 2009 [3]. Previous studies in this region found a significant relation between unmet need for family planning and various socio-economic and demographic factors such as educational level, wealth, maternal age, religion, and media exposure [10–12]. Most studies approach family planning at the individual level however, neglecting possible cluster effects and contextual influences of the community [13–17]. Based on Bronfenbrenner’s social-ecological theory, women’s contraceptive use does not only depend upon individual-level characteristics, but also upon household- and community-level characteristics [18,19]. A study in Malawi revealed that women were less likely to have an unmet need for family planning in communities with a higher share of wealthy households and higher education [20]. Similarly, women in wealthy communities were more likely to use contraceptives than women in poor communities in Ethiopia, regardless of their own household wealth [21]. A multi-level multi-country analysis on contraceptive use found the mean ideal family size to be the most salient community level predictor of modern contraceptive use among young women [19]. Studies also found that women in areas with a lower proportion of health facilities at a convenient distance were less likely to use modern contraceptive methods [22,23]. Women residing in areas with a medium percentage of women complaining about the distance to a health facility were more likely to have unmet need for family planning compared to those in areas with a lower percentage of complainers [20].

In the Democratic Republic of Congo (DRC), unmet need for family planning has been addressed at the individual level in specific regions only, such as Kinshasa or the Vanga health district in Bandundu [24,25]. One study investigated inequalities of the use of modern contraception at the province level in 2007 [26]. To date, no study on the factors associated with unmet need in DRC has been conducted at the national level. Therefore, the objective of this study is to investigate the individual- and community-level factors associated with unmet need for family planning among married women and women living in union aged 15 to 49 in DRC. The aim of this study is twofold. First, we want to verify if a selection of individual and community-level variables significantly influences the likelihood of having unmet needs for family planning in DRC. Second, we want to assess the extent to which the individual variability in unmet need for family planning is due to the variability observed at the community-level, given the individual characteristics. Accurate data on and analysis of unmet need can help Congolese family planning programs target specific sub-groups with various needs, and subsequently help women to prevent unintended pregnancies, reduce unsafe abortions and improve maternal and child health, as well as ensure universal access to sexual and reproductive health-care services.

## Methods

### Study setting

The Democratic Republic of Congo (DRC) is the largest country in Sub-Saharan Africa, by area [27]. In 2012, the total population of DRC was estimated at 77.8 million, with an average growth rate of 3.4 percent [2]. DRC has experienced very high levels of unmet need for family planning for many years, alongside high fertility rates and high maternal and infant mortality rates [2]. Due to decades of continuous political instability, insecurity, violence and external exploitation, the country has not been able to build a stable environment for a fertility transition [2]. Moreover, DRC’s fertility rate increased from 6.3 to 6.6 children per woman between 2007 and 2013 [2].

The population of DRC is characterized by its youth: the proportion of people under 20 years of age is approximately 61% of the country’s total population, 52% of whom are younger than 15 [2]. Poverty is extremely high and widespread: in 2018, it was estimated that 73% of the Congolese population, equaling 60 million people, lived on less than $1.90 a day [27]. The social position of women also remains a concern due to, among other things, socio-cultural constraints that are still strongly rooted in mentalities and which relegate women to the background [2].

The country has made considerable efforts to establish successful family planning programs, but to no avail. In the 1980s, DRC started a promising family planning program (Project des Services des Naissances Desirables), which ended after ten years due to political unrest and economic instability. The years following were characterized by little governmental and political support for family planning and no (from 1991 to 2006) to little financial support from the donor community (i.e., USAID, UNFPA, DFID) [28]. As a result, in 2014, 27.7% of the women of reproductive age (15–49) in DRC had unmet needs for family planning [2]. At the same time, the share of married women whose need for family planning is satisfied with modern methods decreased from 18.4% in 2007 to 16.3% in 2014 in DRC [2]. The modern contraceptive prevalence rate stagnated between 2001 and 2010 between 4.4% and 5.8%, and maternal mortality increased from 543 to 846 deaths per 100.000 live births between 2007 and 2014 [2].

In 2013, DRC became a commitment maker in the Family Planning 2020 program; an international program hosted by the United Nations Foundation aiming to add 120 million new users of modern contraceptives in the world’s 69 poorest countries by 2020. As such, the Congolese Ministry of Public Health claimed to take concrete measures to strengthen family planning by allocating funding to procure contraceptives, equipment, and health supplies to 66 health zones (out of 516 health zones) in the country [25]. However, the distribution of adequate family planning programs is fragmented throughout the provinces and health zones. Less than half (46%) of the country’s 516 health zones dispose of family planning services [29]. The capital Kinshasa has relatively adequate family planning services in both public and private clinics and a higher modern contraceptive prevalence rate, but a large part of the country has limited access to family planning services, particularly in rural areas and conflict-ridden provinces, such as Bas-Uele, Haut-Uele, Bandundu and Equateur [28]. As a result, adolescents and young people–representing 32.8% of the population in 2013 –do not receive the health services they need to address their general or specific sexual reproductive health needs: only 12 out of 516 health zones offer a package of youth-friendly reproductive health activities [2,28]. In addition, the government prohibits abortion under all circumstances, which creates an unsafe environment for women who wish to terminate their pregnancy and leads to a higher incidence of clandestine abortions [30].

### Data and measures

We used cross-sectional data from the 2014 Democratic Republic of Congo Demographic and Health Survey (CDHS), a nationally representative dataset implemented by the Ministry of Monitoring, Planning, and Implementation of the Modern Revolution, in collaboration with the Ministry of Public Health [2]. The CDHS is a multi-stage stratified cluster sample. Results are representative at the level of each of the twenty-six provinces, and each province is an area of study. Within these study areas, three strata were created: the statutory city stratum, the city stratum, and the rural stratum. The final survey unit is the cluster, i.e., the neighborhood or village. The sample consists of 18,360 households in 540 clusters, including 5,474 urban households in 161 clusters and 12,886 rural households in 379 clusters. Within these households, a total of 19,097 women (aged 15–49) were identified, of which 18,827 were successfully interviewed (99% response rate) [2]. In this study, a total of 10,415 women in 536 clusters were included after carrying out the necessary exclusion criteria. Infecund women, non-married women or women not living with a partner, and women who had missing data (0.7%) were excluded.

The dependent variable for this study is the dichotomized variable ‘unmet need for family planning’. It was generated as the sum of the unmet need for spacing and unmet need for limiting, based on the Bradley et al. (2012) definition and estimation method [10]. Women were considered to have an unmet need for family planning when they were not using contraceptives but wanted to space their next birth (spacing) or no longer wanted children (limiting). Unmet need for spacing includes: (a) women at risk of becoming pregnant, who were not using contraception and did not want to become pregnant in the next 2+ years or were undecided whether and when they would like to become pregnant; (b) pregnant women who want the current pregnancy to occur later; and (c) women with postpartum amenorrhea for a period of up to 2 years following a birth that was intended to occur later and who are not using contraception. Unmet need for limiting includes: (a) women at risk of becoming pregnant (i.e., not pregnant, or postpartum amenorrhoeic) who were not using contraception and do not want (more) children; (b) pregnant women whose pregnancy is unintended; and (c) postpartum amenorrhoeic women (for up to two years following the birth of their child) of an unwanted pregnancy who do not use contraception.

The independent variables were assessed at two levels: the individual level and community-level. Individual-level variables include a range of socio-demographic and socio-economic characteristics: woman’s age, educational level, employment status, number of living children, religion, sex household head, knowledge of family planning, media exposure and household wealth. Women’s age was classified into three age groups, representing adolescents (15–24), young women (25–34) and middle-aged women (35–49). Educational attainment was classified into: no education, primary education, and secondary and higher education. Employment status includes two groups: women who are currently employed or have been employed in the 12 months preceding the survey, and women who were not employed in the 12 months preceding the survey. Number of living children was categorized into four groups: no children (0), one or two children (1–2), three to six children (3–6) and seven to thirteen children (7+). Religion was categorized into seven groups, as specified by the CDHS: Catholic, Protestant, other Christian (Revivalist Church), Muslim, Traditional (Vuvamu, Animiste), Others (Salvation Army, Kimbanguist, Bundu dia kongo) and no religion. Sex of household head is classified into male or female. Knowledge of family planning was categorized into three groups: no knowledge of family planning methods, knowledge of modern methods, and knowledge of traditional and folkloric methods. Modern methods include female and male sterilization, hormone- or copper releasing intrauterine devices, injectables, implants, oral contraceptive pills, male and female condoms, vaginal barrier methods (diaphragm, cervical cap and spermicidal foam, jelly, cream, sponge), lactational amenorrhea methods, emergency contraception and other modern methods (contraceptive patch, vaginal ring); traditional and folkloric methods include periodic continence, withdrawal and all other folk methods (usage of herbs, potions, rope and amulets around the hips) [2]. Concerning media exposure, women were considered to be exposed to media if they read a newspaper, watch television or listen to radio at least once a week (“yes”/”no”). The wealth index was used to indicate the socio-economic level of a household. It is based on information on household ownership of specific durable goods (television, radio, car, etc.) and amenities in the house (electricity, type of drinking water supply, type of toilet, flooring material, etc.). The index, generated with principal component analysis, consists of five wealth quintiles: poorest, poorer, average, richer and richest.

Community-level variables were selected based on results of previous studies in other sub-Saharan African countries [19,20,23,31] and created by aggregating individual level data of all community members in a primary sampling unit or cluster as defined by the CDHS. Community women’s educational level was defined as the proportion of women who completed secondary or higher education within a community. Community wealth was defined as the percentage of women living in wealthy (richer or richest wealth-index category) households. Community fertility norms was defined as the percentage of women who had an ideal family size of 6 children or less (the mean ideal family size in DRC). Community distance to health facility was defined as the percentage of women who perceived the distance to a health facility as a big problem. Community media exposure was defined as the percentage of women who were exposed to media. For easy interpretation, the percentages were categorized into “low”, “medium” and “high” based on the tertiles of the community distributions. Place of residence (urban, rural) and province of residence (Kinshasa, Bas-Congo, Bandundu, Equateur, Orientale, Nord-Kivu, Sud-Kivu, Maniema, Katanga, Kasaï Oriental, Kasaï Occidental) were included as well.

### Statistical analyses

All analyses were conducted using STATA version 17.0 (STATA Statistical Software: Release 17, StataCorp LP, College Station, TX). Sample weights were applied to correct for the under- or over-sampling of different strata during sample selection of the CDHS.

The analysis includes both bivariate and multivariate methods. To assess the statistical associations between the outcome (unmet need) and explanatory variables, we first presented the distribution of the respondents by unmet need for spacing, limiting and total family planning by means of absolute and relative frequencies. Pearson’s chi-square tests were used to test for significance. Statistical significance was determined with p-value of 0.05 and less. Second, a two-level mixed effects logistic regression analysis was performed to estimate adjusted odds ratios (aORs) and the extent of random variations between communities. This method was chosen for two reasons. First, DHS data have a hierarchical structure, which means respondents are nested within households and households within clusters, thus women living in the same area may not be independent to one another. Second, the method assesses both the independent fixed effects of the explanatory variable and the community-level random effects on the unmet need for family planning [32].

In total, four models were fitted. Model 0 (null model) included the dependent variable (unmet need) to assess the variance in unmet need between clusters and to estimate the intraclass correlation coefficient. Model I consisted of the dependent variable and the individual-level factors, Model II consisted of the dependent variable and the community-level factors, and Model III included the dependent variable and both the individual- and community-level factors. The fixed effects (measures of association), expressed as adjusted odds ratios with 95% confidence intervals, estimate the associations between the dependent and independent variables. The log of the odds of unmet need for family planning was defined as $\log\left(\frac{{\text{Y}}_{\text{ij}}}{\left(1-{\text{Y}}_{\text{ij}}\right)}\right)=\beta_{\text{0}}+\beta_{\text{1}}{\text{X}}_{\text{ij}}+\beta_{\text{2}}{\text{Z}}_{\text{ij}}+\mu_{\text{0j}}+{\text{e}}_{\text{ij}}$, where: Y_ij_ is the odds of having unmet need for family planning for the ith women in the jth community; 1 − Y_ij_ is the probability of not having unmet need for family planning for the ith women in the jth community; β_0_ is the intercept coefficient; β_1_ and β_2_ indicate the fixed coefficients; X_ij_ and Z_ij_ refer to individual- (age, educational level, number of living children, religion, media exposure, knowledge of family planning, wealth index) and community-level variables (province, place of residence, community educational level, community fertility preference, community media exposure, community wealth and community distance to health facility); μ_0j_ is the random error at the community level and e_ij_ the random error at the individual level.

The random effects (measures of variation) were assessed using the intraclass correlation coefficient (ICC), proportional change in variance (PCV) and the median odds ratio (MOR). To estimate the goodness of fit of the final model in comparison to the preceding models, we used the log-likelihood test. The ICC indicates the extent to which community-level factors explain the total variation in unmet need for family planning in the null model. It indicates whether the variation in unmet need for family planning is primarily within or between communities. It was calculated as $\text{ICC}=\frac{\sigma_{\mu 0}^{2}}{\sigma_{\mu 0}^{2}+\frac{\pi^{2}}{3}}$, where σ^2^ is the variance between communities and $\frac{\pi^{2}}{3}$ represents the variance within communities (≈3.29). The PCV measures the total variation attributed to individual-level factors and community level factors. It was calculated using the formula $\text{PCV}=\frac{{\text{V}}_{\text{e}}-{\text{V}}_{\text{mi}}}{{\text{V}}_{\text{e}}}$, where V_e_ represents the community variance in the null model and V_mi_ the community variance in the subsequent models. MOR calculates the median value of the odds ratio between the community with the highest risk at unmet need for family planning and the community with the lowest risk at unmet need for family planning, randomly picked out. MOR shows the extent to which the individual probability of having unmet need for family planning is determined by the community individuals reside in. It was calculated as $\text{MOR}=(\exp\sqrt{2\sigma^{2}*0.6745})\approx \exp\;(0.95\sigma^{2})$, where σ^2^ is the variance at the community-level and 0.6745 is the 75th centile of the cumulative distribution function of the normal distribution with mean 0 and variance 1 [33]. The goodness-of-fit was assessed with the Akaike information criterion (AIC). The lowest AIC represents the most fit model.

## Results

### Characteristics of the respondents

**Fig 1:**
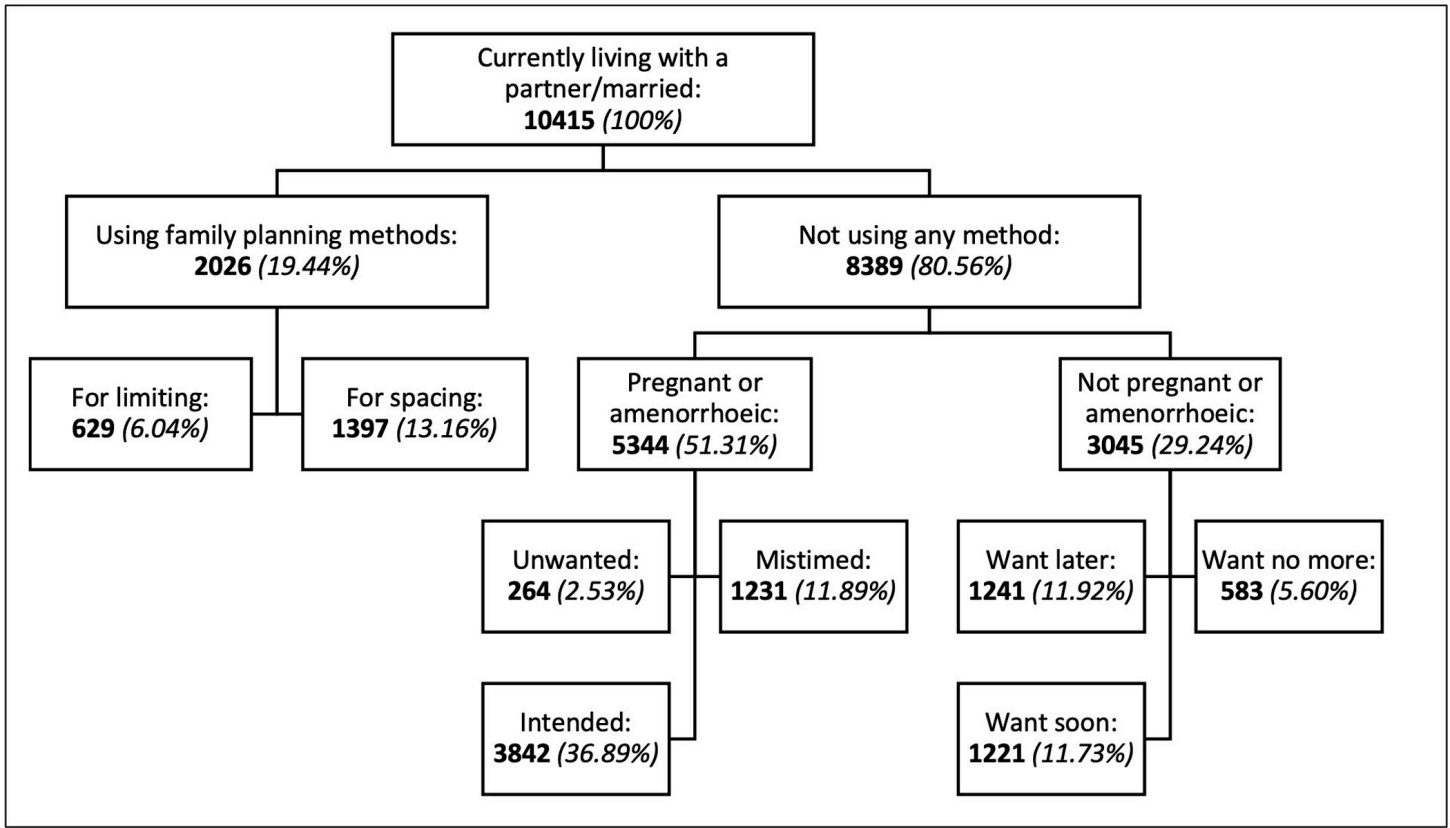
Unmet need among women of reproductive age who are married or living with a partner in DRC, 2014.

The final sample of this study included 10,415 women, nested in 536 clusters. As shown in Fig 1, a total of 3,328 women (31.94%) had unmet need for family planning, of which 847 women (8.13%) for limiting and 2,481 women (23.81%) for spacing.

The distribution of the respondent’s characteristics according to unmet need are shown in Table 1A and 1B. The proportion of women who had unmet need for family planning was highest among women who were respectively oldest (35–49) (35.72%), with primary education (32.78%), who are employed (34.28%), who had seven or more children (44.34%), were not religious (37.89%), who had a female household head (34.68%), who were exposed to media (32.23%), who had knowledge of modern methods (32.11%), and who live in rich households (36.10%). At the community level, the proportion of women who had unmet need for family planning was highest among women in communities with a high proportion of women with at least secondary education (33.33%), with a medium proportion of women exposed to media (34.67%), with a medium proportion of women in rich households (36.13%), with a high proportion of women wanting six or less children (35.07%) and with a high proportion of women who perceived the distance to a health facility as problematic (32.71%). Finally, the proportion of women who had unmet need for family planning was highest among women living in the provinces Orientale (39.81%) and Equateur (39.77%), and among women living in urban areas (33.65%).

**Table 1:** a. Prevalence of unmet need for family planning among married or in union women (n = 10415), individual-level factors. b. Prevalence of unmet need for family planning among married or in union women (n = 10415), community-level factors.

	Unmet need for spacing (n = 2481)	Unmet need for limiting (n = 847)	Total unmet need (n = 3328)	P-value
**Individual-level factors**				
**Age** 15–24 25–34 35–49	900 (28.56%) 1176 (25.72%) 405 (15.02%)	43 (1.36%) 246 (5.38%) 558 (20.70%)	943 (29.93%) 1422 (31.10%) 963 (35.72%)	P = 0.000
**Educational level** No education Primary Secondary or higher	465 (22.05%) 1103 (24.26%) 913 (24.25%)	187 (8.87%) 387 (8.51%) 273 (7.25%)	652 (30.92%) 1490 (32.78%) 1186 (31.50%)	P = 0.245
**Employment** Employed Unemployed **Number of living children** 0 1–2 3–6 7+	533 (27.65%) 1946 (22.93%) 122 (16.07%) 951 (28.35%) 1211 (23.76%) 197 (16.27%)	128 (6.64%) 718 (8.46%) 6 (0.79%) 32 (0.95%) 469 (9.20%) 340 (28.08%)	661 (34.28%) 2664 (31.39%) 128 (16.86%) 983 (29.31%) 1680 (32.97%) 537 (44.34%)	P = 0.014 P = 0.000
**Religion** No religion Catholic Protestant Other Christian Muslim Traditional Others **Sex household head** Male Female	25 (26.32%) 662 (24.21%) 738 (24.39%) 892 (23.09%) 42 (21.54%) 13 (24.53%) 109 (24.01%) 2090 (23.60%) 391 (25.02%)	11 (11.58%) 283 (10.35%) 236 (7.80%) 272 (7.04%) 13 (6.67%) 4 (7.55%) 28 (6.17%) 696 (7.86%) 151 (9.66%)	36 (37.89%) 945 (34.19%) 974 (32.19%) 1164 (30.13%) 55 (28.21%) 17 (32.08%) 137 (30.18%) 2786 (31.46%) 542 (34.68%)	P = 0.006 P = 0.012
**Media exposure** No Yes	1879 (23.64%) 602 (24.34%)	652 (8.20%) 195 (7.89%)	2531 (31.85%) 797 (32.23%)	P = 0.724
**Knowledge of family planning**				P = 0.003
No knowledge Folkloric/traditional Modern methods	225 (25.31%) 83 (18.57%) 2173 (23.92%)	75 (8.44%) 28 (6.26%) 744 (8.19%)	300 (33.75%) 111 (24.83%) 2917 (32.11%)	
**Wealth index** Poorer Poor Average Rich Richer	631 (23.95%) 519 (22.08%) 535 (24.50%) 471 (26.24%) 325 (22.34%)	177 (6.72%) 174 (7.40%) 197 (9.02%) 177 (9.86%) 122 (8.38%)	808 (30.66%) 693 (29.48%) 732 (33.52%) 648 (36.10%) 447 (30.72%)	P = 0.000
**Community-level factors**				
**Province** Kinshasa Bandundu Bas-Congo Equateur Kasaï Oriental Kasaï Occidental Katanga Maniema Nord-Kivu Orientale Sud-Kivu	131 (20.06%) 354 (24.77%) 100 (20.88%) 468 (28.68%) 175 (18.36%) 293 (23.02%) 247 (19.90%) 147 (26.06%) 145 (26.61%) 288 (26.30%) 133 (23.92%)	53 (8.12%) 125 (8.75%) 40 (8.35%) 166 (10.17%) 41 (4.30%) 60 (4.71%) 88 (7.09%) 46 (8.16%) 65 (11.93%) 134 (12.24%) 29 (5.22%)	184 (28.18%) 479 (33.52%) 140 (29.23%) 634 (38.85%) 216 (22.67%) 353 (27.73%) 335 (26.99%) 193 (34.22%) 210 (38.53%) 422 (38.54%) 162 (29.14%)	P = 0.000
**Place of residence** Urban Rural	791 (24.80%) 1690 (23.37%)	293 (9.19%) 554 (7.66%)	1084 (33.99%) 2244 (31.03%)	P = 0.000
**Community education level (sec+)** Low Med High	1,375 (23.33%) 628 (24.49%) 478 (24.36%)	455 (7.72%) 216 (8.42%) 176 (8.97%)	1,830 (31.05%) 844 (32.92%) 654 (33.33%)	P = 0.081
**Community media exposure (exp)** Low Med High	1,777 (23.63%) 529 (25.58%) 175 (21.01%)	594 (7.90%) 188 (9.09%) 65 (7.80%)	2,371 (31.53%) 717 (34.67%) 240 (28.81%)	P = 0.003
**Community wealth index (rich+)** Low Med High	1,628 (23.32%) 310 (25.22%) 543 (24.57%)	515 (7.38%) 134 (10.90%) 198 (8.96%)	2,143 (30.70%) 444 (36.13%) 741 (33.53%)	P = 0.000
**Community fertility preference (6-)** Low Med High	584 (19.84%) 1,119 (26.11%) 778 (24.38%)	129 (4.38%) 377 (8.80%) 341 (10.69%)	713 (24.22%) 1,496 (34.91%) 1,119 (35.07%)	P = 0.000
**Distance to health facility (problem)** Low Med High	997 (24.13%) 831 (23.43%) 653 (23.81%)	327 (7.92%) 276 (7.78%) 244 (8.90%)	1,324 (32.05%) 1,107 (31.21%) 897 (32.71%)	P = 0.439

Source: Democratic Republic of Congo Demographic and Health Survey (2013–2014).

### Multilevel logistic regression analysis

Table 2 presents the fixed effects by means of odds ratios with a 95% confidence interval. Model I contained individual-level factors only. The results revealed that woman’s age, sex of head of household and number of living children were significantly associated with unmet need for family planning. Educational level, employment status, religion, media exposure, knowledge of family planning and wealth index showed no significant results. Compared to adolescent women (15–24), all women older than 24 had significantly lower odds of unmet need for family planning. Young women (25–34) were 24% less likely to have unmet need for family planning (aOR = 0.76, CI: 0.64–0.90), while middle-aged women (35–49) were 3% less likely to have unmet need (aOR = 0.66, CI: 0.52–0.83). Furthermore, the odds of having unmet need significantly increased with the number of living children, with an aOR of 2.42 (CI: 1.77–3.30) for women with one to two children to an aOR of 6.28 (CI: 4.18–9.42) for women with seven children or more. Women living in households with a female household head also had higher odds of having unmet need (aOR = 1.21, CI: 1.04–1.41). Finally, although not significant, the results revealed that all religious women were less likely to have unmet needs as compared to non-religious women.

**Table 2:** Two-level logistic analysis of individual- and community level factors associated with total unmet need among married or in union women.

	Model 0	Model I AOR (95% CI)	Model II AOR (95% CI)	Model III AOR (95% CI)
**Individual level**				
**Age** 15–24 25–34 35–49		1 0.76** (0.64–0.90) 0.66*** (0.52–0.83)		1 0.75*** (0.63–0.90) 0.65*** (0.51–0.82)
**Educational level** No education Primary Secondary and higher		1 1.06 (0.88–1.26) 0.99 (0.81–1.21)		1 1.05 (0.87–1.26) 0.96 (0.78–1.18)
**Employment status** Unemployed Employed **Number of living children** 0 1–2 3–6 7+		1 0.88 (0.74–1.04) 1 2.43*** (1.78–3.30) 3.26*** (2.32–4.58) 6.28*** (4.18–9.43)		1 0.91 (0.76–1.07) 1 2.46*** (1.81–3.35) 3.32*** (2.37–4.66) 6.46*** (4.28–9.73)
**Sex of head of household** Male Female **Religion** No religion Catholic Protestant Other Christian Muslim Traditional Others		1 1.21* (1.04–1.41) 1 0.89 (0.48–1.64) 0.76 (0.43–1.34) 0.70 (0.39–1.26) 0.54 (0.26–1.12) 0.73 (0.33–1.59) 0.87 (0.45–1.68)		1 1.22* (1.04–1.42) 1 0.84 (0.45–1.54) 0.72 (0.40–1.28) 0.70 (0.39–1.27) 0.50 (0.24–1.05) 0.80 (0.37–1.77) 0.89 (0.46–1.72)
**Media exposure** No Yes		1 1.00 (0.85–1.19)		1 1.00 (0.84–1.19)
**Knowledge of family planning** Modern methods Folkloric/traditional No knowledge		1 1.13 (0.74–1.73) 1.02 (0.77–1.35)		1 1.17 (0.77–1.79) 1.00 (0.75–1.34)
**Wealth index** Poorer Poor Average Richer Richer		1 0.89 (0.75–1.05) 1.10 (0.92–1.31) 1.16 (0.92–1.44) 0.99 (0.77–1.28)		1 0.90 (0.76–1.07) 1.11 (0.93–1.34) 1.08 (0.83–1.40) 0.88 (0.60–1.28)
**Community level**				
**Province** Kinshasa Bandundu Bas-Congo Equateur Kasaï Occidental Kasaï Oriental Katanga Maniema Nord-Kivu Orientale Sud-Kivu			1 1.60* (1.08–2.38) 1.21 (0.81–1.78) 2.11*** (1.46–3.03) 1.05 (0.67–1.63) 1.26 (0.84–1.88) 1.31 (0.90–1.91) 1.61* (1.02–2.52) 2.06*** (1.38–3.08) 1.88*** (1.30–2.72) 1.21 (0.77–1.90)	1 1.35 (0.90–2.04) 1.03 (0.69–1.55) 1.82** (1.24–2.68) 0.85 (0.54–1.33) 1.05 (0.69–1.58) 1.05 (0.71–1.55) 1.49 (0.92–2.40) 1.66* (1.10–2.55) 1.60* (1.10–2.32) 0.96 (0.60–1.54)
**Place of residence** Urban Rural			1 0.96 (0.73–1.27)	1 0.97 (0.73–1.28)
**Community education level** Low Med High			1 0.93 (0.78–1.12) 0.89 (0.66–1.19)	1 0.95 (0.79–1.15) 0.98 (0.72–1.33)
**Community media exposure** Low Med High			1 0.89 (0.73–1.08) 0.88 (0.57–1.34)	1 0.91 (0.75–1.11) 0.94 (0.62–1.44)
**Community wealth index** Low Med High			1 1.38**(1.08–1.77) 1.47* (1.05–2.05)	1 1.32* (1.01–1.72) 1.46° (0.98–2.18)
**Community fertility preference** Low Med High			1 1.44*** (1.18–1.75) 1.42** (1.10–1.84)	1 1.45*** (1.20–1.76) 1.43** (1.10–1.86)
**Distance to health facility** Low Med High			1 1.06 (0.89–1.25) 1.06 (0.87–1.29)	1 1.07 (0.90–1.25) 1.06 (0.86–1.29)

°P<0.065,

*P < 0.05,

**P < 0.01,

***P < 0.001. Source: Democratic Republic of Congo Demographic and Health Survey (2013–2014).

In Model II, the community-level factors province, community wealth index and community fertility preference were significantly associated with unmet need for family planning. Women from the province Bandundu (aOR = 1.60, CI: 1.08–2.38), Equateur (aOR = 2.11, CI: 1.46–3.03), Maniema (aOR = 1.61, CI: 1.02–2.52), Nord-Kivu (aOR = 2.06, CI: 1.38–3.08) and Orientale (aOR = 1.88, CI: 1.30–2.72) all have significantly higher odds of unmet need for family planning compared to women from Kinshasa. All other provinces showed a higher unmet need as well, but results are not significant. Women from communities with a medium (aOR = 1.38, CI: 1.08–1.77) and high (aOR = 1.47, CI: 1.05–2.05) proportion of women in wealthy households were more likely to have unmet need for family planning compared to those in communities with a low proportion of wealthy households. Finally, women in communities with a medium (aOR = 1.44, CI: 1.18–1.75) and high (aOR = 1.42, CI: 1.10–1.84) proportion of women with an ideal family size of 6 children or less had significantly higher odds of having unmet need for family planning compared to those in communities with a low proportion of women who have an ideal family size of 6 children or less.

The final model generally generates the same patterns, except for a few changes. Age and sex household head show similar results, but number of living children generates more significant differences in model III, with higher odds for women with one to two children (aOR = 2.46, CI: 1.81–3.35), women with three to six children (aOR = 3.32, CI: 2.37–4.66), and women with seven or more children (aOR = 6.46, CI: 4.28–9.73). On the community level, all provinces have slightly lower odds of having unmet need for family planning compared to the preceding model. The odds for the provinces Equateur (aOR = 1.82, CI: 1.24–2.68), Nord-Kivu (aOR = 1.66, CI: 1.10–2.55) and Orientale (aOR = 1.60, CI: 1.10–2.32) remained significant, while Bandundu and Maniema did not show significant results anymore. Community wealth and community fertility preference showed similar results in both models. Except for religion, all other variables without a significant correlation do not show notable results in model III. Their odds range between 0.90 and 1.07, hence do not have a strong effect on unmet need for family planning.

In additional analysis we examined if the association between place of residence and unmet need for family planning was modified by household wealth. The addition of an interaction term in the multivariable models showed insignificant results, thus it was not included in the models.

Table 3 presents the random effects by means of the intra class coefficient (ICC), community variance and proportional change in variance (PVC). In the null model, the ICC revealed that 9.39% of the total variance in unmet need for family planning was explained by between-community variation of the variables. The ICC declined in subsequent models to 7.19% in the final model. The final ICC indicates low between-community variability: individuals within communities were no more similar to each other than individuals from different communities. In Model I the PCV indicates that only 0.5% of the between-community variance in unmet need for family planning was explained by the individual level characteristics. This small change in the estimate of the between-community variance is suggesting that the distribution of the individual-level variables is similar across communities. The final model’s PCV suggests that 25.4% of the between-community variance in unmet need for family planning was explained by the combined variables at the individual- and community levels. The goodness of fit was determined with the Akaike’s Information Criterion (AIC). The final model has the lowest AIC (11969.33), hence was considered the best fit model.

**Table 3:** Random effects of two-level logistic analysis of individual- and community level factors associated with total unmet need for family planning among married or in union women.

Random effects				
ICC	9.39%	9.35%	7.20%	7.19%
Community Var (95% CI)	0.34 (0.27–0.43)	0.34 (0.27–0.43)	0.26 (0.20–0.33)	0.26 (0.19–0.33)
MOR	1.75 (1.64–1.87)	1.74 (1.64–1.87)	1.62 (1.53–1.73)	1.62 (1.52–1.73)
PCV	Ref	0.5%	25.1%	25.4%
Model fit				
Log-likelihood	-6111.016	-5984.571	-6067.021	-5939.665
AIC	12226.03	12015.14	12180.04	11969.33

ICC = Intra-Class Correlation; Community var = Community variance; MOR = Median Odds Ratio; PCV = Proportional Change in Variance; AIC = Akaike’s Information Criterion, ref = reference group. Source: Democratic Republic of Congo Demographic and Health Survey (2013–2014).

## Discussion and conclusion

The purpose of this study was to investigate the prevalence and factors associated with unmet need for family planning in DRC among married women of reproductive age. In our final sample of 10,415 women, the total unmet need for family planning was 31.94%, of which 8.13% had unmet need for limiting and 23.81% for spacing. By means of a two-level mixed-effect logistic regression analysis, the results revealed that both individual- and community level factors influence the probability of having unmet need for family planning. Apart from the individual socio-demographic characteristics woman’s age, number of living children, and sex of head of household, unmet need for family planning was significantly influenced by factors on the community level, including province, community wealth and community fertility preference.

First, results indicated a negative relationship between unmet need and age: unmet need for family planning decreased with increasing age. Compared to adolescent women (15–24), all women aged 25 and older were less likely to have unmet need for family planning. This result, confirmed in earlier studies [14,15,21,34], is consistent with the general hypothesis that unmet need is most prominent among adolescents, as they face more obstacles to obtain contraceptives [35]. Although in general, the desire for later childbearing is becoming more common in sub-Saharan Africa and adolescents express their wish to delay, space or limit births, many are not using contraception [35–37]. Different factors have been put forward to explain this pattern: fear that confidentiality will not be respected in clinics, stigma associated with early intercourse, low empowerment to negotiate contraceptive use and family planning with -often older- partners, and lack of education or resources which may limit their ability to access the information and services they need [9,35–39].

Second, our results revealed the importance of sex of the household head, as women in female headed households had higher odds of unmet need. Similar studies set in sub-Saharan Africa find both higher [17], and lower [40] odds of unmet need for women in female headed households. This result prompts for further research, as women in these positions are often considered to be the decision makers with regards to health issues [41]. However, the higher unmet need could also be due to other barriers to contraceptive use, such as poor access to family planning services, socio-cultural constraints, or stigma [1,42,43].

Furthermore, our results showed that the higher the number of living children, the higher the likelihood of an unmet need for family planning. This finding is in line with other studies in sub-Saharan Africa as well [15,17,21]. Women with a higher parity, whether desired or not, probably are more inclined to use family planning methods as they have reached their ideal family size. Yet, many of them lack knowledge, access, or recourses to use family planning methods. Our findings also revealed that women in communities with a medium or high proportion of women with an ideal family size of 6 children or less were respectively 43% and 52% more likely to have unmet need compared to women from communities with a low proportion of women who have an ideal family size of 6 children or less. A study on women from rural areas in Rwanda and Nepal shows similar results: a strong negative relationship between the community fertility preference and the use of contraceptives [23]. Our results support the hypothesis based on Bronfenbrenner’s socio-ecological theory in that community-level characteristics influence contraceptive use at the individual-level [18,19].

Province of residence appeared to be an important factor as well. Compared to women from Kinshasa, women from Equateur had the highest odds of unmet need for family planning, followed by women from Orientale and Nord-Kivu. Equateur, Orientale, and Nord-Kivu all occupy the northern area of DRC. This region lacks adequate and developed health infrastructure to address reproductive health; the health centers and health posts are dependent on medical supplies from international organizations, which frequently results in shortages of medicines, equipment, and other materials [26,44]. Moreover, Nord-Kivu and Orientale are both scenes of ongoing political instability and conflict [45,46]. The higher likelihood of unmet need could therefore also be related to poor access to health-care services, as many areas remain inaccessible and alien to government and international humanitarian interventions [26]. Efforts should be made to make health care and family planning services more accessible in these conflict-ridden regions.

Finally, this study revealed that women residing in communities with a medium and high proportion of women from wealthy households were more likely to have unmet needs compared to women from communities with a low proportion of women from wealthy households. Our results both correspond to [3,17] and contradict results from other similar studies [34,47]. Women from communities with a higher proportion of wealthy households could be more inclined to use family planning methods for delaying, limiting, or spacing, but family planning programs are not able to meet the demand for family planning methods [3]. This observation is worthy of further investigation, as it could indicate the onset of a fertility transition, where fertility preferences in wealthier (and urban) areas tend to decline first.

### Research and policy implications

Our results highlight the need for family planning programs specifically focused on and adapted to adolescents (15–24) as this age group has the highest unmet need of all age groups. DRC has an extremely young population with an estimated 61% of people under 20 years of age, therefore targeting this age group could benefit a large number of people [9]. It is critical to expand access to family planning services, especially to the most vulnerable groups of adolescents: those who are out of school, have little or no education, and/or live in poverty [35].

Further research on unmet need for family planning and contraceptive use in DRC is needed. Particularly qualitative research, which could provide a more comprehensive understanding of why unmet need for family planning is as high among certain groups of women: those from wealthier communities and communities with a higher ideal family size. Improving family planning programs would not only help increase contraceptive uptake in all communities but could also decrease the number of unsafe abortions and consequently reduce maternal mortality, as it is estimated about 7% of maternal deaths in sub-Saharan Africa were related to abortion [48]. Referring to the 2030 Sustainable Development Goals, promoting family planning programs and ensuring universal access to sexual and reproductive health and reproductive rights will in general benefit gender equality and the empowerment of women and girls in DRC.

### Strengths and limitations

The use of the Demographic and Health Survey dataset generates some limitations for this study. Data was obtained by means of a cross-sectional study, which makes it impossible to establish causal relations. Furthermore, the DHS survey obtained self-reported retrospective information. This may implicate recall bias; thus, the interpretation of the results needs to be done with caution. The use of the DHS is also a major strength of this study, as it is nationally representative and therefore generalizable to the whole country. Furthermore, we used the most recent DRC DHS dataset and worked with a large population (n = 10415). Another strength of this study is the use of a multilevel mixed effects logistic regression analysis to accommodate the hierarchical nature of the DHS data. The inclusion of community-level variables proved to be very insightful: both community wealth and community fertility preferences are important factors associated with unmet need for family planning.

## Supporting information

S1 File (DOCX)
